# 4 weeks of high-intensity interval training does not alter the exercise-induced growth hormone response in sedentary men

**DOI:** 10.1186/2193-1801-3-336

**Published:** 2014-07-02

**Authors:** Hiroto Sasaki, Takuma Morishima, Yuta Hasegawa, Ayaka Mori, Toshiaki Ijichi, Toshiyuki Kurihara, Kazushige Goto

**Affiliations:** Graduate School of Sport and Health Science, Ritsumeikan University, Shiga, Japan; Faculty of Sport and Health Sciences, Ritsumeikan University, 1-1-1, Nojihigashi, Kusatsu, Shiga 525-8577 Japan

**Keywords:** Endocrine response, Ectopic fat, Fat metabolism, Intensive exercise, Obesity

## Abstract

This study determined the effects of high-intensity interval training on the exercise-induced growth hormone (GH) responses, whole body and regional fat content. Twenty-four sedentary males were randomized to either a high-intensity interval training (HIT) group or a low-intensity continuous training (LT) group. The HIT group performed intermittent exercises at 85% of , whereas the LT group performed continuous exercise for 22 min at 45% of . Before and after 4 weeks of training, hormonal and metabolic responses to acute exercise were determined. Acute exercise significantly increased GH concentrations in both groups (*p* < 0.05). However, the responses did not change after training period in either group. Furthermore, the training did not significantly affect intramyocellular or intrahepatic lipid content in either group. The present study indicates that 4 weeks of high-intensity interval training does not alter the exercise-induced GH responses, whole body fat mass or intramyocellular and intrahepatic lipid content in sedentary males.

## Introduction

Growth hormone (GH) has been shown to affect substrate oxidation pattern and body composition, because GH promotes lipolysis and fat oxidation (Gravholt et al. 1999). Administration of GH for 2 wk increases 24-h energy expenditure and fat oxidation (Hansen et al. 2005). GH secretion is also influenced by a variety of physiological conditions, including age, gender, nutrition status, body composition, and physical fitness level (Salvadori et al. 2010). Exercise, in particular, is a potent stimulus of GH secretion (Gilbert et al. 2008; Goto et al. 2007; Kanaley et al. 1997). The magnitude of exercise-induced GH secretion is dependent on the intensity (Pritzlaff et al. 1999) and duration of exercise (Wideman et al. 2006).

The accumulation of whole body and visceral fat mass strongly attenuates GH secretion (Makimura et al. 2008). Spontaneous (Eliakim et al. 2006; Kanaley et al. 1999) and exercise-induced (Rasmussen et al. 2008; Weltman et al. 2008) GH responses are impaired in obese people with marked abdominal fat accumulation compared with responses in people of normal weight. Furthermore, growing evidence suggests that accumulated fat in non-adipose tissue (ectopic fat) impairs GH secretion. For example, it was reported that the GH response to GHRH and arginine administration was attenuated by the accumulation of intramyocellular lipid (IMCL) (Misra et al. 2008). In addition, a previous study reported that intrahepatic lipid content (IHL) was negatively correlated with the peak GH concentration following GHRH and arginine administration (Bredella et al. 2009).

The increment of GH concentration during exercise is associated with the facilitation of lipolysis during the post-exercise period (Enevoldsen et al. 2007), and a positive correlation between peak GH concentration during exercise and fat oxidation during recovery was reported (Pritzlaff et al. 2000). Thus the exercise-induced GH secretion may have a favorable influence in terms of weight loss. Although GH secretion is generally attenuated in obese subjects, a great reduction in body weight caused by long-term caloric restriction increases GH secretion (Rasmussen et al. 2008). By contrast, a recent study demonstrated that moderate aerobic training for 4 weeks augmented the exercise-induced GH response with minimal or no weight loss in obese subjects (Salvadori et al. 2010), suggesting that aerobic training may be an effective tool for increasing GH secretion. Furthermore, considering that the exercise-induced GH response is dependent on exercise intensity, repetition of high-intensity interval training (HIT) may elicit greater improvement of the exercise-induced GH response compared with lower-intensity training.

Therefore, the purpose of the present study was to investigate the effects of HIT for 4 weeks on exercised-induced GH secretion. We also determined changes in whole body fat mass, abdominal fat area, IMCL and IHL, which influence GH secretion. We hypothesized that HIT for 4 weeks would increase the magnitude of the exercise-induced GH response.

## Methods

### Subjects

Twenty-four healthy sedentary males participated in this study. Their height, weight and body mass index (BMI) were 172.5 ± 1.4 cm, 71.2 ± 2.0 kg, and 23.9 ± 0.5 kg/m^2^, respectively (mean ± standard error [SE]). All subjects were informed about the experiment procedure and purpose of this study. Their written informed consent was obtained subsequently. The study was approved by the Ethics Committee for Human Experiments at Ritsumeikan University, Japan.

#### Study design and experimental protocol

After completing baseline measurements, subjects were randomized to either the high-intensity interval training group (HIT group; n = 12) or low-intensity continuous training group (LT group; n = 12). None of the subjects were participating in any regular training program at the start of the experiment. There were no significant differences in baseline measurements regarding physical characteristics between the groups (Table 1). Subjects participated in the training program for 4 weeks (3 days/week, 12 sessions total). Although several types of HIT have been previously used (e.g., repeated bouts of 30s maximal pedaling; Burgomaster et al. 2006; Gibala and McGee 2008 we selected submaximal HIT model in term of practical application among normal people. Similar exercise protocol has been applied in a previous study for patients with type 2 diabetes (Little et al. 2011). Specifically, the HIT consisted of 10 sets of 1 min pedaling at 85% of maximal oxygen uptake ( ) with a 30 s rest between sets. The LT consisted of 22 min of continuous pedaling at 45% of , based on results of a preliminary study designed to ensure that energy expenditure during exercise was the same in both regimens. At the end of 2 weeks during training period, we measured  again to adjust workload in accordance with improvement of aerobic capacity (Data not shown). Heart rate (HR) was measured continuously using a wireless HR monitor (Polar RS400™, Polar Electro Japan Co, Tokyo, Japan) in all training sessions. The rating of perceived exertion (RPE) was determined during the first and final training sessions (Borg 1973). Aerobic capacity and body composition were evaluated before and after training periods. We also determined hormonal and metabolic responses to acute exercise (30 min of continuous pedaling at 60% of ) before and after training periods.

**Table 1:** **Physical and metabolic parameters of subjects before and after training period**

		Before training	After training
Body weight (kg)	HIT	73.4 ± 2.8	73.7 ± 2.9
	LT	69.0 ± 2.7	69.4 ± 2.8
BMI (kg/m^2^)	HIT	24.3 ± 0.7	24.4 ± 0.7
	LT	23.4 ± 0.8	23.6 ± 0.8
Percent fat (%)	HIT	21.4 ± 1.2	20.9 ± 1.3
	LT	19.5 ± 1.4	19.9 ± 1.4
(ml/min)	HIT	3031 ± 144	3255 ± 117*
	LT	2854 ± 146	2991 ± 162*

Data are means ± SE. *P < 0.05 versus before training.

### Measurements before and after training periods

#### Maximal oxygen uptake

 was assessed before and after training periods using a graded power test on a cycle ergometer (AEROBIKE 75XLIII, COMBI WELLNESS Co, Tokyo, Japan). The test began at 80 W and the load increased progressively in 30-W increments every 2 min until exhaustion. The test was terminated when the subject failed to maintain the prescribed pedaling frequency of 80 rpm or reached the plateau of . Respiratory gases were collected and analyzed using an automatic gas analyzer (AE300S, Minato Medical Science Co., Ltd, Tokyo, Japan). Data were averaged every 30 s. HR was measured continuously using a wireless HR monitor (Polar RS400™, Polar Electro Japan Co, Tokyo, Japan).

### Whole body fat mass and abdominal fat area

Whole body and regional fat masses were determined before and after training periods. Whole body fat mass was evaluated using the bioimpedance method (In Body 720, BIOSPACE Co., Tokyo, Japan). Subcutaneous fat area of the abdomen (SFA) and visceral fat area (VFA) were measured with a 1.5-T magnetic resonance (MR) system (SignaHDxt 1.5 T GE Health Care UK Ltd, Buckinghamshire, England). During MR scans, the subject remained in the supine position. A series of transaxial MRI scans for the abdomen was acquired with a respiratory trigger (field of view 420 × 420 mm, slice thickness 10 mm, no gap, TE = 7.3 ms, TR = one respiration). SFA and VFA were measured at the slice between L3 and L4 (L: Lumbar spine). All measurements were carried out by the same investigator using specially designed image analysis software (SliceOmatic 4.3, Tomovision Inc., Magog, Canada).

### IMCL and extramyocellular lipid content (EMCL)

IMCL and EMCL were measured using a 1.5-T MR system (GE Healthcare Japan Co., Ltd, Tokyo, Japan) with an eight-channel body array coil. The subject’s right thigh was positioned parallel to the main magnetic field. Multi-slice T1-weighted axial spin-echo images (TR/TE 600/7.8 ms, thickness 10 mm, FOV 440 × 440 mm, matrix size 256 × 256) were initially acquired to guide the positioning of the volume of interest. Thereafter, single voxel proton magnetic resonance spectroscopy (^1^H-MRS) measurements were performed using the Point Resolved Spectroscopy (PRESS) sequence (TR/TE 2000/35 ms, 2 × 2 × 2 cm^3^, 32 acquisitions). The voxel was placed on the middle of the right vastus lateralis muscle at the midpoint between the greater trochanter and knee cleft. The voxel was placed carefully to avoid including visible interstitial tissue, subcutaneous fat or blood vessels. The spectrum was acquired without water suppression. Absolute concentrations of IMCL and EMCL were evaluated using LCModel software (version 6.3; S. Provencher, PhD, Oakville, Ontario, Canada) and the customized calculation reported by a previous study (Weis et al. 2009). The peak chemical shift of IMCL was adjusted to 1.3 ppm, and that of EMCL was adjusted to 1.5 ppm.

### IHL content

IHL content was measured by a 1.5-T MR system (SignaHDxt 1.5 T GE Health Care UK Ltd, Buckinghamshire, England) with an eight-channel body array coil. Transverse images of the liver were used to ensure accurate positioning of the voxel. A single voxel ^1^H-MRS was performed using the free-breathing PRESS sequence with a respiratory trigger (TE = 26 ms, TR = two respirations, 2 × 2 × 2 cm^3^, 16 acquisitions; 2048 data points over 2500 Hz spectral width). The voxel was placed within the posterior right lobe of the liver visually free from hepatic portal vein, gallbladder and fatty tissue. Post-processing of spectroscopic data was performed, and the combined liver lipid peaks (0.9, 1.3 and 2.0-2.2 ppm) were detected by a customized algorithm using LCModel software. Briefly, data were automatically scaled to an unsuppressed water peak (4.7 ppm), and the lipid content to the sum of water, and the lipid ratio (percent) were expressed. Abdominal transverse MRI images and spectra used to estimate IMCL, EMCL and IHL contents were obtained in the morning after an overnight fast.

### Assessment of hormonal and metabolic responses to acute exercise

**Figure 1:**
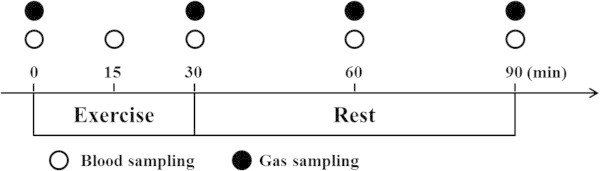
**Exercise test protocols for blood sampling and respiratory measurements.**

Before and after training periods, subjects arrived at the laboratory in the morning after fasting for ≥ 12 h. The post-training visit was scheduled at least 48 h after the end of the training period to exclude acute effects of the final training session. After resting for 30 min, a polyethylene catheter was inserted into an antecubital vein and a baseline blood sample was obtained. Subjects subsequently performed 30 min of a pedaling exercise at 60% of  with a cycle ergometer (exercise period, 0-30 min). After completing the exercise, subjects rested on a comfortable chair for 60 min (recovery period, 30-90 min). Venous blood samples were collected every 15 min during the exercise period and every 30 min during the recovery period to determine hormonal and metabolite responses to acute exercise (Figure 1).

Serum and plasma samples were prepared by 10 min of centrifugation and stored at -80°C until analysis. From baseline samples, concentrations of blood glucose, serum cholesterol (HDL or LDL cholesterol), triglyceride (TG), free fatty acids (FFA), glycerol, GH, insulin, and cortisol were measured. Blood samples during exercise and the recovery period were used to determine concentrations of blood glucose, serum FFA, glycerol, GH, insulin, and cortisol.

Serum GH and cortisol concentrations were measured using radioimmunoassay (RIA) at a clinical laboratory (SRL, Inc., Tokyo, Japan). The intra-assay CVs after measurement were 2.6% for serum GH and 4.9% for serum cortisol. Serum insulin concentrations were also measured using a chemiluminescent enzyme immunoassay at the clinical laboratory. The intra-assay CV after measurement was 3.4% for serum insulin. Serum glycerol concentrations were determined in duplicate using a commercially available kit (Cayman Chemical Company, MI, USA). The intra-assay CV was 2.1% for serum glycerol. Serum FFA concentrations were measured using a commercially available enzymatic colorimetric kit (NEFA-HRII, Wako Pure Chemical Industries, Osaka, Japan). The intra-assay CV was 1.4% for serum FFA. Serum TG concentrations were measured using an enzymatic method at a clinical laboratory (SRL, Inc., Tokyo, Japan). The intra-assay CV was 2.6% for serum TG. Serum cholesterol concentrations were determined using ultraviolet absorption spectrophotometry at a clinical laboratory (SRL, Inc, Tokyo, Japan). The intra-assay CV was 1.4% for serum cholesterol.

Respiratory gases were collected to determine , carbon dioxide production ( ) and ventilation volume during exercise and recovery periods. All measurements were performed in each 4-min period simultaneously with blood sampling, except for blood samples obtained at 15 min (Figure 1). The respiratory exchange ratio (RER) was calculated from  and  and used to estimate the relative contribution of fat (% fat contribution) and carbohydrate oxidation (% carbohydrate contribution) to total energy expenditure (Manetta et al. 2002). The percentage fat and carbohydrate contributions were calculated without measuring urinary nitrogen because of the negligible contribution of protein to substrate oxidation during exercise (Beidleman et al. 2002). Appropriate calibrations of  and  sensors and the volume transducer were conducted prior to the start of exercise. HR was monitored continuously during exercise and recovery periods. The RPE was determined every 15 min during exercise.

### Physical activity and diet survey

Subjects were advised to maintain their normal physical activity and diet throughout the experimental period. They wore an acceleration sensor (Actimarker, Panasonic Electric Works Co, Osaka, Japan) for 1 week, and daily physical activity was calculated from the obtained data. Daily energy intake was calculated using a brief-type self-administered diet history questionnaire (BDHQ) (Kobayashi et al. 2012). The BDHQ data were analyzed by an expert dietitian.

### Statistical analyses

Data are expressed as means ± SE for all variables. To compare body composition, physical fitness and baseline blood data between before and after training periods, a two-way (group [HIT, LT] × period [before and after training periods]) analysis of variance (ANOVA) with repeated measures was conducted. When ANOVA revealed a significant interaction or a main effect, post hoc tests were performed to assess where the difference occurred. Hormonal and metabolic responses to acute exercise were initially analyzed using a two-way (period [before and after training periods] × time [0, 15, 30, 60, 90 min]) ANOVA with repeated measures for each group and subsequent post hoc tests. Delta values of hormonal responses were compared between before and after training periods using a paired *t*-test. *P* < 0.05 was considered to indicate a significant difference.

## Results

### Physical characteristics and cardiorespiratory fitness

Table 1 shows physical characteristics and  before and after training periods. There were no differences in body composition or  between the two groups before the training period. Body weight, BMI and percentage fat did not change significantly in either group between before and after training periods.  increased significantly in both groups after the training period (HT; 8.3 ± 2.6%, LT; 4.7 ± 1.3%, compared with pre-training values, *p* < 0.05). However, there was no significant difference in the percentage change between groups.

### Fasting blood samples

There were no differences in serum TG, HDL-cholesterol and LDL-cholesterol concentrations between HIT (TG; 72 ± 8 mg/dl, HDL-cholesterol; 51 ± 2 mg/dl, LDL-cholesterol; 105 ± 6 mg/dl) and LT (TG; 80 ± 10 mg/dl, HDL-cholesterol; 53 ± 4 mg/dl, LDL-cholesterol; 88 ± 6 mg/dl) groups before the training period. These concentrations did not change significantly after the training period in either group.

### Abdominal fat area

Table 2 shows abdominal fat area before and after training periods. Before the training period, VFA and SFA were not significantly different between groups. VFA and SFA did not change significantly after the training period in either group. Consequently, abdominal fat area, which was calculated by the sum of SFA and VFA, was not significantly different between before and after training periods in either group.

**Table 2:** **Abdominal fat area before and after training period**

		Before training	After training
Subcutaneous fat area (cm^2^) (SFA)	HIT	153 ± 16	160 ± 19
	LT	132 ± 13	132 ± 12
Visceral fat area (cm^2^) (VFA)	HIT	59 ± 9	57 ± 4
	LT	50 ± 6	52 ± 8
Abdominal fat area (cm^2^)	HIT	211 ± 17	216 ± 20
	LT	183 ± 17	183 ± 16

Values are means ± SE. Abdominal fat area indicates the sum of sucutaneous and visceral fat area.

### IMCL and IHL content

Table 3 shows IMCL, EMCL and IHL content before and after training periods. Before the training period, IMCL, EMCL and IHL contents were not significantly different between the groups. These values did not change significantly after the training period in either group.

**Table 3:** **Intramyocellular lipid and intrahepatic lipid content before and after training period**

		Before training	After training
IMCL (mmol/kg)	HIT	12.5 ± 2.4	14.2 ± 2.5
	LT	9.7 ± 1.2	10.4 ± 1.7
EMCL (mmol/kg)	HIT	17.8 ± 3.4	20.5 ± 3.2
	LT	24.0 ± 5.0	22.3 ± 3.8
IHL (arbitrary unit)	HIT	44.5 ± 4.7	46.6 ± 4.3
	LT	49.9 ± 6.2	46.8 ± 7.2

Values are means ± SE. IMCL; intramyocellular lipid content, EMCL; extracellular lipid content, IHL; intrahepatic lipid content.

### Hormonal responses to acute exercise

**Figure 2:**
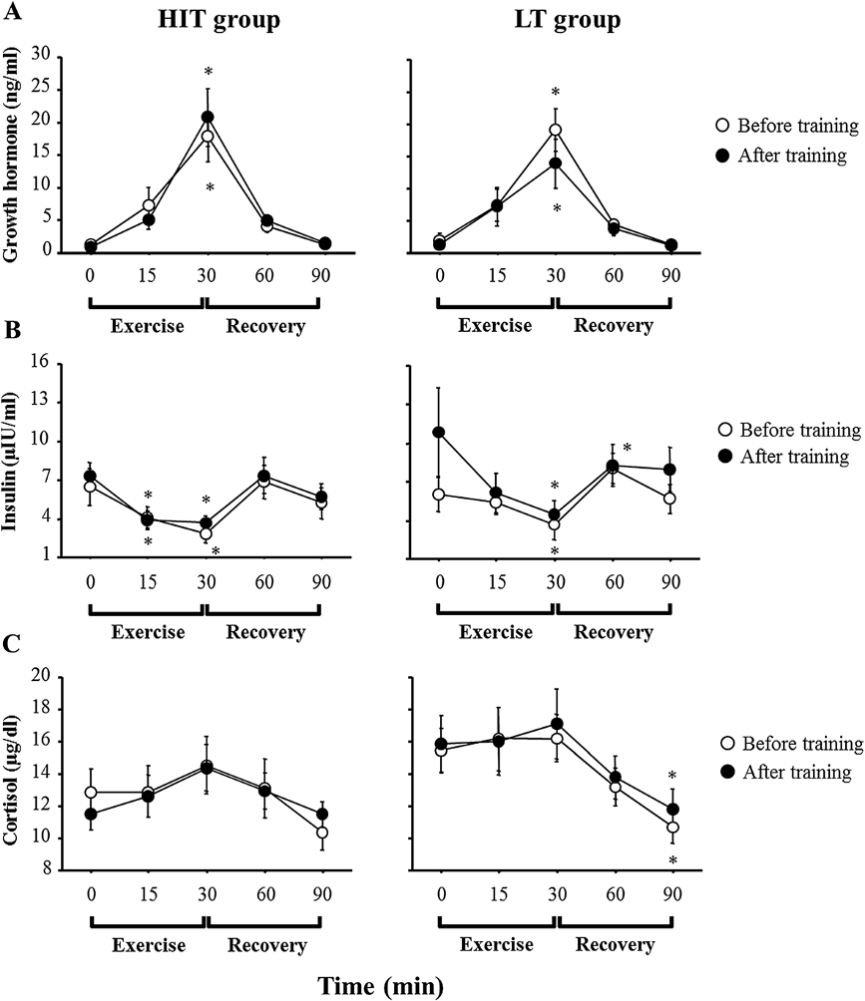
**Hormonal responses to the exercise.** **(A)** Growth hormone **(B)** Insulin **(C)** Cortisol. Values represent the means ± SE. *Significantly different than 0 min (*p* < 0.05).

Figure 2 shows the time course of changes in serum GH, insulin and cortisol concentrations during exercise and recovery periods. Before the training period, serum GH concentrations increased significantly during exercise in both groups (*p* < 0.05). Although a significant increase in serum GH concentration was found during exercise in both groups after the training period, the responses were not significantly different between before and after training periods in either group (Figure 2A). Serum insulin concentrations decreased significantly during exercise in both groups (*p* < 0.05). Although a significant decrease in serum insulin concentration was revealed after the training period, the responses were not significantly different between before and after training periods in either group (Figure 2B). Serum cortisol concentrations did not change significantly during exercise in either group, but decreased significantly during the recovery period in the LT group. Serum cortisol responses to exercise were similar between before and after training periods in both groups (Figure 2C). The area under the curve (AUC) of serum GH, insulin and cortisol concentrations during exercise and recovery periods did not change significantly between before and after training periods in either group.

**Figure 3:**
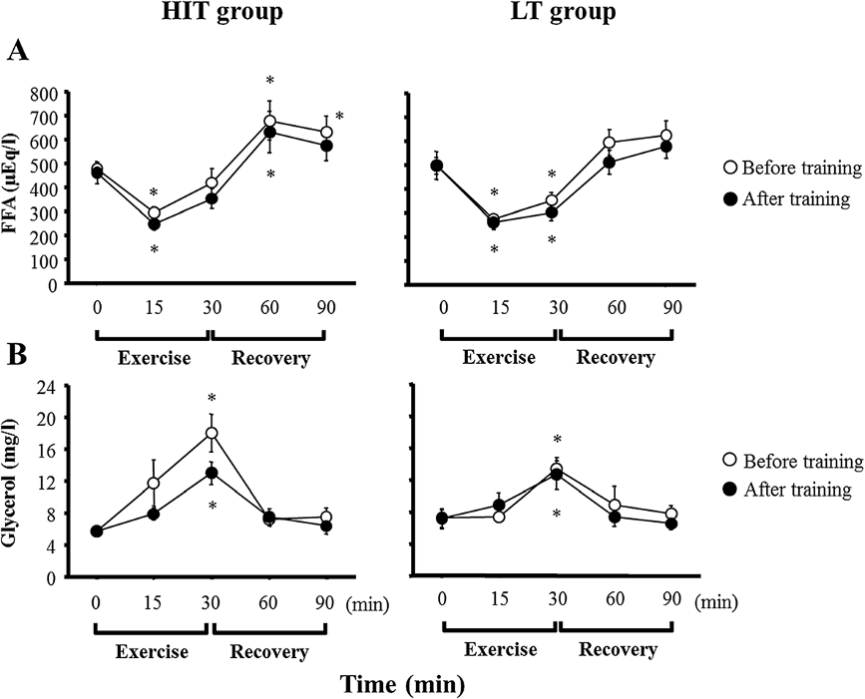
**Serum FFA and glycerol responses to the exercise.** **(A)** FFA **(B)** Glycerol. Values represent the means ± SE. *Significantly different than 0 min (*p* < 0.05).

Figure 3 shows serum FFA and glycerol concentrations during exercise and recovery periods. Serum FFA concentrations decreased significantly during exercise in both groups (*p* < 0.05). However, serum FFA responses were similar between before and after training periods in both groups (Figure 3A). Before the training period, serum glycerol concentrations increased significantly during exercise in both groups (*p* < 0.05). Although serum glycerol concentrations increased significantly during exercise after training, the responses were not significantly different between before and after training periods in either group (Figure 3B). The AUCs of serum FFA and glycerol concentrations did not change significantly between before and after training periods in either group.

### Cardiorespiratory response to acute exercise

Table 4 shows the cardiorespiratory and substrate oxidation responses during exercise and recovery periods. Before the training period, HR increased significantly during exercise in both groups (*p* < 0.05). After the training period, the peak HR during exercise tended to be lower than before training in the HIT group (*p* = 0.067).

**Table 4:** **Cardiorespiratory and substrate oxidation responses to 30 min exercise before and after training period**

Time			0 min	30 min	60 min	90 min
Heart rate (bpm)	HIT	Before	75 ± 5	163 ± 5*	91 ± 3*	82 ± 3
		After	73 ± 3	155 ± 4*	97 ± 4*	81 ± 4*
	LT	Before	73 ± 3	167 ± 4*	93 ± 3*	86 ± 3*
		After	70 ± 3	160 ± 4*	99 ± 3*	85 ± 2*
(ml/min)	HIT	Before	260 ± 14	2041 ± 101*	269 ± 19	269 ± 12
		After	253 ± 15	2157 ± 107*	269 ± 17	268 ± 20
	LT	Before	258 ± 11	1953 ± 99*	276 ± 15	261 ± 15
		After	243 ± 17	1953 ± 120*	247 ± 11	251 ± 14
(ml/min)	HIT	Before	211 ± 13	1924 ± 102*	195 ± 15	210 ± 12
		After	200 ± 13	2056 ± 97*	205 ± 12	208 ± 15
	LT	Before	211 ± 9	1892 ± 101*	206 ± 11	203 ± 11
		After	207 ± 18	1919 ± 116*	192 ± 12	198 ± 12
RER	HIT	Before	0.81 ± 0.02	0.94 ± 0.01*	0.73 ± 0.02*	0.78 ± 0.02
		After	0.79 ± 0.01	0.96 ± 0.01*	0.77 ± 0.03	0.78 ± 0.02
	LT	Before	0.83 ± 0.01	0.97 ± 0.01*	0.75 ± 0.01*	0.78 ± 0.02*
		After	0.85 ± 0.02	0.99 ± 0.01*	0.77 ± 0.02*	0.79 ± 0.02*
Carbohydrate contribution (%)	HIT	Before	35.4 ± 7.3	79 ± 3.9*	11.9 ± 4.0*	23.2 ± 5.9
		After	26.8 ± 3.2	84.6 ± 2.8*	21.5 ± 8.8	25.5 ± 6.7
	LT	Before	39.7 ± 4.7	88.3 ± 2.4*	12.9 ± 3.5*	24.5 ± 5.2*
		After	47.5 ± 5.1	91.9 ± 2.0*	21.5 ± 6.4*	27.6 ± 4.9*
Fat contribution (%)	HIT	Before	64.6 ± 7.3	20.2 ± 3.9*	88.1 ± 3.9*	76.8 ± 5.9
		After	73.2 ± 3.2	15.4 ± 2.8*	78.5 ± 8.8	74.5 ± 6.7
	LT	Before	60.4 ± 4.7	11.8 ± 2.4*	87 ± 3.5*	75.6 ± 5.2*
		After	52.5 ± 5.1	8.2 ± 2.0*	78.5 ± 6.4*	72.4 ± 4.9*

Values are means ± SE. *; *P* < 0.05 vs. 0 min. Carbohydrates and fat contributions indicate the relative utilization rate of energy resource to the total energy expenditure during 30 min exercise and 60 min recovery.

 and  during exercise and recovery periods were not significantly different between before and after training periods in either group. In addition, there were no significant differences in RER during exercise and recovery periods between before and after training periods in either group. Furthermore, the percentage carbohydrate or percentage fat contributions did not change significantly between before and after training periods in either group.

### Physical activity and diet survey

Before the training period, estimated daily energy expenditure was not significantly different between groups (HIT group, 2331 ± 83 kcal/day vs. LT group, 2341 ± 67 kcal/day). These values did not change significantly after the training period in either group (HIT group, 2234 ± 68 kcal/day vs. LT group, 2290 ± 81 kcal/day).

Dietary energy intake estimated by BDHQ showed no significant difference between groups (HIT group, 2659 ± 262 kcal/day vs. LT group, 2261 ± 258 kcal/day) before the training period. No significant change was observed in dietary energy intake after the training period in either group (HIT group, 2375 ± 209 kcal/day vs. LT group, 2058 ± 271 kcal/day).

## Discussion

To our knowledge, this is the first study to determine the effects of HIT on the exercise-induced GH response and ectopic fat accumulation. Our results indicate that the exercise-induced GH response was unaffected by 4 weeks of HIT. Additionally, there were no significant changes in IMCL or IHL between before or after high- or low-intensity training.

High-intensity anaerobic exercise (e.g., resistance exercise, sprint exercise) generally evokes a large increase in GH concentration after exercise (Gilbert et al. 2008; Collier et al. 2006; Stokes et al. 2002). However, previous studies have demonstrated that resistance training for 3 (Zajac et al. 2010) or 8 weeks (Kraemer et al. 1998) does not change the magnitude of resistance exercise-induced GH secretion. Furthermore, it was reported that 6 weeks of sprint training decreased the GH response to exercise (Stokes et al. 2004). By contrast, a recent study (Salvadori et al. 2010) revealed that 4 weeks of moderate-intensity aerobic training significantly increased the GH response to the same exercise, suggesting that exercise mode affects effects of training on the exercise-induced GH response.

In contrast to our hypothesis, the GH response to a single bout of exercise was not significantly different between before and after 4 weeks of low- or high-intensity training. There are several plausible reasons for the absence of changes in the GH response in the HIT group. First, the subjects in this study had relatively high GH responses to exercise at pre-training compared with those of obese subjects (Kanaley et al. 1999), which may influence the present results. Second, the accumulation of VFA, IMCL or IHL, which inhibit GH secretion (Makimura et al. 2008; Misra et al. 2008; Bredella et al. 2009), was not significantly different between before and after training periods in either group. Although aerobic training for longer duration substantially reduces VFA (Johnson et al. 2009; Heydari et al. 2012), the training program used in our study, which consisted of a relatively small training volume (14.5-22 min/session) and short duration (4 weeks), may be insufficient to alter exercise-induced GH secretion. In the present study, we have specifically focused on HIT, because growing evidences indicate that HIT promotes oxidative capacity in working muscles similar to traditional prolonged training (Burgomaster et al. 2008; Little et al. 2010; Hood et al. 2011). However, data from high-intensity continuous training group would be helpful to clarify impact of energy expenditure during exercise.

IMCL and IHL were measured using ^1^H-MRS before and after training periods, and no significant differences were found in either group. Aerobic training has previously shown to reduce IMCL in patients with type 2 diabetes (Tamura et al. 2005). By contrast, a previous study reported that 4 weeks of aerobic training without caloric restriction did not affect IMCL in obese individuals (Johnson et al. 2009), consistent with the present results. The inconsistent observation for IMCL in earlier studies can be explained by differences in experimental design, including whether the aerobic training was conducted in combination with caloric restriction. Information concerning the effect of exercise training on IHL content is limited, whereas IHL content has been shown to be reduced by caloric restriction alone and by exercise together with caloric restriction (Tamura et al. 2005; Shah et al. 2009; Vitola et al. 2009). Although several studies have shown that aerobic training without caloric restriction reduced IHL accumulation (Johnson et al. 2009; Keating et al. 2012; Bacchi et al. 2013), the effect of exercise training alone without caloric restriction on IHL content is not consistent among previous studies (Devries et al. 2008). Therefore, aerobic training combined with caloric restriction would be expected to reduce IMCL or IHL and subsequently improve GH secretion. Interestingly, in the present study, the exercise-induced GH response did not correlate with either IMCL or IHL in all subjects (n = 24), but IHL content was inversely correlated with the exercise-induced GH response in obese subjects (r = -0.865, *p* < 0.01, n = 8). Therefore, IHL accumulation seems to inhibit the exercise-induced GH response in a manner dependent on body composition.

Exercise-induced GH secretion augments fat metabolism during the post-exercise period (Enevoldsen et al. 2007; Wee et al. 2005). Thus, we hypothesized that the enhanced GH response following 4 weeks of HIT would increase exercise-induced lipolysis and fat oxidation. However, the hypothesis was not supported by our results because the time course of changes in serum glycerol and RER did not change significantly after 4 weeks of HIT. Moreover, insulin or cortisol responses to exercise, which also affect fat metabolism, were similar between before and after training periods in both groups.

The aim of this study was to determine the effect of HIT on the exercise-induced GH response in combination with detailed changes of whole body and regional fat content. However, our study had several limitations; we recruited healthy sedentary subjects with less fat accumulation and a greater exercise-induced GH response compared with obese people. Clearly, it was difficult to detect changes in endocrine regulations by training intervention. Therefore, further investigations using obese people are essential.

In conclusion, 4 weeks of HIT did not affect the magnitude of the exercise-induced GH response. In addition, the training intervention did not significantly change abdominal fat or IMCL or IHL content.
